# Sun leaves up-regulate the photorespiratory pathway to maintain a high rate of CO_2_ assimilation in tobacco

**DOI:** 10.3389/fpls.2014.00688

**Published:** 2014-12-03

**Authors:** Wei Huang, Shi-Bao Zhang, Hong Hu

**Affiliations:** Key Laboratory of Economic Plants and Biotechnology, Kunming Institute of Botany, Chinese Academy of SciencesKunming, China

**Keywords:** CO_2_ assimilation, light acclimation, photorespiratory pathway, RuBP carboxylation, RuBP regeneration

## Abstract

The greater rate of CO_2_ assimilation (*A*_n_) in sun-grown tobacco leaves leads to lower intercellular and chloroplast CO_2_ concentrations and, thus, a higher rate of oxygenation of ribulose-1,5-bisphosphate (RuBP) than in shade-grown leaves. Impairment of the photorespiratory pathway suppresses photosynthetic CO_2_ assimilation. Here, we hypothesized that sun leaves can up-regulate photorespiratory pathway to enhance the *A*_n_ in tobacco. To test this hypothesis, we examined the responses of photosynthetic electron flow (*J*_T_) and CO_2_ assimilation to incident light intensity and intercellular CO_2_ concentration (*C*_i_) in leaves of ‘k326’ tobacco plants grown at 95% sunlight (sun plants) or 28% sunlight (shade plants). The sun leaves had higher photosynthetic capacity and electron flow devoted to RuBP carboxylation (*J*_C_) than the shade leaves. When exposed to high light, the higher Rubisco (ribulose-1,5-bisphosphate carboxylase/oxygenase) content and lower *C*_i_ in the sun leaves led to greater electron flow devoted to RuBP oxygenation (*J*_O_). The *J*_O_/*J*_C_ ratio was significantly higher in the sun leaves than in the shade leaves under strong illumination. As estimated from CO_2_-response curves, the maximum *J*_O_ was linearly correlated with the estimated Rubisco content. Based on light-response curves, the light-saturated *J*_O_ was linearly correlated with light-saturated *J*_T_ and light-saturated photosynthesis. These findings indicate that enhancement of the photorespiratory pathway is an important strategy by which sun plants maintain a high *A*_n_.

## INTRODUCTION

In natural habitats, plants are subject to temporal and spatial variations in light intensity. Many species modulate the biochemical composition and morphology of leaves or whole plants to acclimate to their light environments (Terashima and Hikosaka, 1995; Niinemets et al., 1998; Terashima et al., 2005; Yamori et al., 2010a). In general, the leaves of plants grown under high light (sun leaves) have higher levels of cytochrome *f* (Cyt *f* ), ATP synthase, Rubisco, and other Calvin Cycle enzymes (Evans, 1987; Terashima and Evans, 1988; Hikosaka, 1996; Hikosaka and Terashima, 1996; Yamori et al., 2010a). Those leaves that acclimate to more intense light usually have higher capacities for electron transport and CO_2_ assimilation (Yamori et al., 2010a). Timm et al. (2012) have reported that over-expression of the H-protein of glycine decarboxylase (a key enzyme in the photorespiratory pathway) leads to considerably increased net photosynthesis in *Arabidopsis thaliana*, suggesting that enhancement of photorespiratory pathway potentially improves the *A*_n_. However, the relationship between photorespiration and photosynthesis in plants acclimated to high light is unclear.

In some species, the greater *A*_n_ by plants grown under and adapted to high light means that they have lower intercellular and chloroplast CO_2_ concentrations than those acclimated to low light (Hanba et al., 2002; Oguchi et al., 2005; Yamori et al., 2010a). When the chloroplast CO_2_ concentration is low, the specificity of Rubisco to O_2_ increases and then induces a rise in the rate of oxygenation of RuBP. Under such conditions, the higher Rubisco content in sun leaves accelerates the rate of RuBP oxygenation. During RuBP oxygenation, one molecule of glycolate-2-phosphate and one of glycerate-3-phosphate are formed (Ogren, 1984). Glycolate-2-phosphate cannot be used by plants for biosynthetic reactions and is a potential inhibitor of chloroplast functioning (Anderson, 1971). Therefore, it must be converted into glycerate-3-phosphate through the photorespiratory pathway (Leegood et al., 1995). To avoid this side effect of glycolate-2-phosphate, it is speculated that the capacity of photorespiratory pathway is greater in sun leaves than in shade leaves. Results from previous studies have shown that the effect of the light environment on the capacity of that pathway is controversial. For example, growth irradiance can influence photorespiration in leaves from *Arisaema heterophyllum* and *Swietenia*, but not from *Dipteryx* (Muraoka et al., 2000; Marenco et al., 2001).

According to the C_3_ photosynthesis model, the *A*_n_ is limited by both RuBP carboxylation and RuBP regeneration (Farquhar et al., 1980). In both sun and shade leaves of tobacco plants grown with high nitrogen supply, the *A*_n_ at *C*_a_ and high light tends to be limited by RuBP regeneration (Yamori et al., 2010a). Because the Calvin Cycle intermediate glycerate-3-phosphate is critical for RuBP regeneration, impairment of the recycling of glycolate-2-phosphate into glycerate-3-phosphate depletes RuBP regeneration and, ultimately, depresses the *A*_n_ (Somerville and Ogren, 1980, 1981, 1983; Takahashi et al., 2007). Therefore, we hypothesize that up-regulation of the photorespiratory pathway may be an important strategy by which plants grown under high light can accelerate RuBP regeneration and subsequently maintains the high rate of photosynthesis.

The aim of this study was to investigate further the role of the photorespiratory pathway in photosynthesis. Plants of *Nicotiana tabacum* were grown at 24/18°C (day/night) under either 95 or 28% sunlight. The *A*_n_ and *J*_T_ to incident light intensity were evaluated at 24°C and a CO_2_ concentration of 400 μmol mol^-1^. Those same parameters were also examined in response to incident CO_2_ concentration when plants were exposed to 24°C and 1200 μmol photons m^-2^ s^-1^. Our results indicated that sun leaves indeed up-regulate the photorespiratory pathway to maintain a high photosynthesis rate.

## MATERIALS AND METHODS

### PLANT MATERIALS AND GROWTH CONDITIONS

Seedlings of the ‘k326’ cultivar of tobacco (*Nicotiana tabacum*) were cultivated in plastic pots, and then transferred to a phytotron at Kunming Institute of Botany, Yunnan, China (elevation 1900 m, 102°41′E, 25°01′N). Conditions included day/night temperatures of 24°C/18°C, 60% relative air humidity, and a constant *C*_a_ of 400 μmol mol^-1^. Sunlight was used as the source of illumination in the phytotron. Sun plants received approximately 95% of full sunlight (maximum intensity at noon ≈1990 μmol photons m^-2^s^-1^). To establish shade conditions, we added a layer of netting over other plants to reduce photosynthetic active radiance to approximately 28% of full sunlight (maximum intensity ≈580 μmol photons m^-2^s^-1^). During the experimental period (24 October to 24 December 2013), none of the plants experienced any water or nutrient stresses. After 50 days, the mature leaves that had been produced since transplanting were chosen for photosynthetic measurements.

### CHLOROPHYLL FLUORESCENCE AND GAS EXCHANGE MEASUREMENTS

An open gas exchange system incorporating infrared CO_2_ and water vapor analyzers (Li-6400XT; Li-Cor Inc., Lincoln, NE, USA) was used to determine the *A*_n_ in the phytotron. During the measurement period, the relative air humidity was 60% and the air temperature was 24°C. To generate a light response curve, the leaves of both sun and shade plants were exposed to high light (i.e., 1200 μmol photons m^-2^ s^-1^) for 20 min to obtain a steady state. Afterward, photosynthetic parameters were evaluated every 2 min at a controlled *C*_a_ of 400 μmol mol^-1^ and photosynthetic photon flux densities(PPFDs) of 2000, 1600, 1200, 800, 500, 300, 200, 100, 50, 20, or 0 μmol photons m^-2^ s^-1^. The CO_2_ assimilation rate versus *C*_i_ was measured (von Caemmerer and Farquhar, 1981) at 1200 μmol photons m^-2^ s^-1^. For each *A*/*C*_i_ curve, photosynthetic rate reached a steady state at 400 μmol mol^-1^, subsequently decreased to a lower limit of 50 μmol mol^-1^ and then increased stepwise to an upper limit of 2000 μmol mol^-1^. Each stepwise measurement was completed within 2–3 min. Using those *A*/*C*_i_ curves, we calculated the maximum rates of RuBP regeneration (*J*_max_) and RuBP carboxylation (*V*_cmax_) according to the method of Long and Bernacchi (2003). The leaf Rubisco content was estimated according to the empirical equation of Yamori et al. (2010a) as *y* = 35.3*x* + 6.6, where *y* is *V*_cmax_ (μmol CO_2_ m^-2^ s^-1^) and *x* is Rubisco content (μmol m^-2^).

Chlorophyll fluorescence was measured simultaneously with gas exchange measurements using a fluorometer chamber (6400-40; Li-Cor Inc.). The fluorescence parameters *F_s_* and *F_m_*′ were determined as previously described (Baker and Rosenqvist, 2004), with *F_s_* representing the steady fluorescence and *F_m_*′ the maximum fluorescence after light-adaptation. The effective quantum yield of PSII was calculated as Φ_PSII_ = (*F_m_*′ –* F_s_*)/*F_m_*′ (Genty et al., 1989).

### ESTIMATING THE RATE OF PHOTOSYNTHETIC ELECTRON FLOW

The total *J*_T_ through PSII (*J*_T_) was calculated as *J*_T_ = Φ_PSII_ × PPFD × 0.85 × 0.5 (Krall and Edwards, 1992). Because leaf absorbance (*L_abs_*) in tobacco differs little between sun and shade leaves (Miyake et al., 2005), we assumed here that *L_abs_* was 0.85 in both types. The constant of 0.5 was used based on the assumption of an equal distribution of photons between photosystems I and II (Miyake et al., 2005). The light saturation point (LSP) is the PPFD that causes 95% of the maximum *A*_n_ while the light compensation point (LCP) is the PPFD under which the net photosynthetic rate is 0. If CO_2_ assimilation is limited in the leaves, the water–water cycle cannot eliminate excess excitation energy by acting as a major alternative electron sink (Driever and Baker, 2011). Therefore, we allocated the electron flow through PSII to RuBP carboxylation (*J*_C_) and oxygenation (*J*_O_). These were estimated according to the method of Valentini et al. (1995):

$$J_{O}=2/3\times \left(J_{T}-4\times \left(A_{n}+R_{d}\right)\right)$$

$$J_{C}=1/3\times \left(J_{T}+8\times \left(A_{n}+R_{d}\right)\right)$$

where *A*_n_ is CO_2_ assimilation and *R*_d_ represents the rate of mitochondrial respiration. We estimated *R*_d_ from the linear region of the light response curve between PPFDs of 20 and 100 μmol photons m^-2^ s^-1^ (Zhang et al., 2009, 2013).

### STATISTICAL ANALYSIS

The results were displayed as mean values of four independent measurements. Data were subjected to independent *t*-test using the SPSS 16.0 for windows (SPSS Inc., Chicago, IL, USA). A level of *P* < 0.05 was used to determine whether differences were significant between sun and shade leaves.

## RESULTS

**FIGURE 1:**
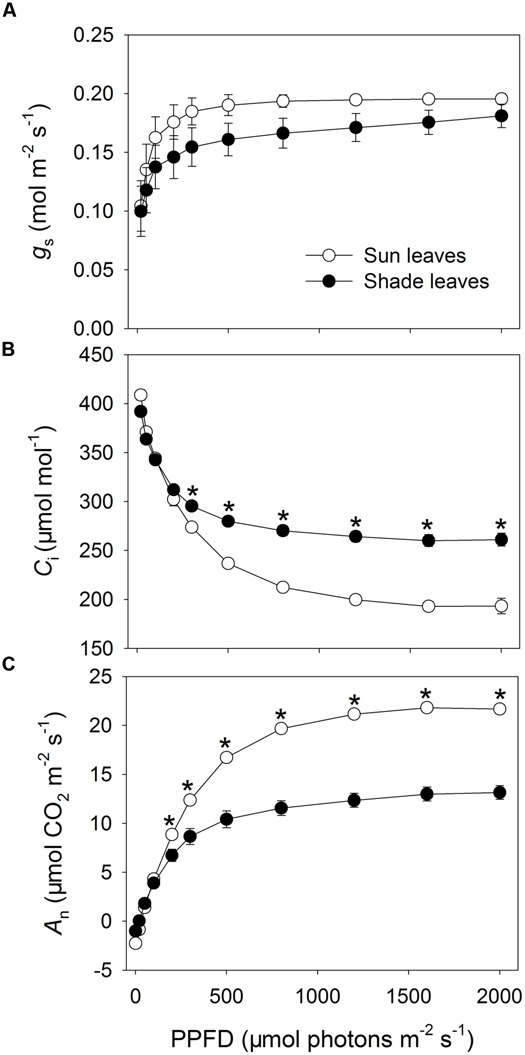
**Responses of *g*_**s**_ (**A**), *C*_**i**_ (**B**), and CO_**2**_ assimilation (*A*_**n**_, **C**) to incident photosynthetic photon flux density (PPFD) for sun and shade leaves of tobacco.** Values are means ± SE (*n* = 4). Significant differences between leaf types (indicated by asterisks) were examined by independent *t*-test (*P* < 0.05).

Compared with the shade plants, the sun plants had significantly higher values for LSP, LCP, apparent quantum efficiency, and saturating photosynthetic rate (**Table 1**) as well as relatively higher *g*_s_ than the shade leaves (**Figure 1A**). When exposed to light intensities above 300 μmol photons m^-2^ s^-1^, values for *C*_i_ were significantly lower in sun leaves (*P* < 0.0001; **Figure 1B**). At 1200 μmol photons m^-2^ s^-1^, the *C*_i_ was 193 and 261 μmol mol^-1^ in the sun and shade leaves, respectively. When light intensities were above 200 μmol photons m^-2^ s^-1^, the sun leaves showed a higher *A*_n_ (*P* < 0.0001; **Figure 1C**). Under strong illumination, i.e., 2000 μmol photons m^-2^ s^-1^, photosynthetic rate was 21.8 and 13.1 μmol CO_2_ m^-2^ s^-1^ in sun and shade leaves, respectively. These results indicated that the sun leaves generally had greater photosynthetic capacity, which led to their lower *C*_i_ values.

**Table 1:** Parameters describing photosynthetic CO_**2**_ assimilation.

	Sun leaves	Shade leaves	Significance
LSP (μmol photons m^-2^ s^-1^)	1164 ± 48.9	797 ± 18.5	0.0004
LCP (μmol photons m^-2^ s^-1^)	32 ± 2.8	20 ± 3.6	0.04
Apparent quantum efficiency	0.0685 ± 0.003	0.046 ± 0.003	0.0001
Saturating photosynthetic rate (μmol CO_2_ m^-2^ s^-1^)	23.8 ± 0.5	13.6 ± 0.8	0.0001

*The light saturation point (LSP) is the PPFD at which the *A*_n_ is 95% of the maximum, and the light compensation point (LCP) is the PPFD under which the net photosynthetic rate is 0. Data are mean values ± SD (*n* = 4). Significant differences between sun and shade leaves were examined by independent *t*-tests (*P* < 0.05).*

**FIGURE 2:**
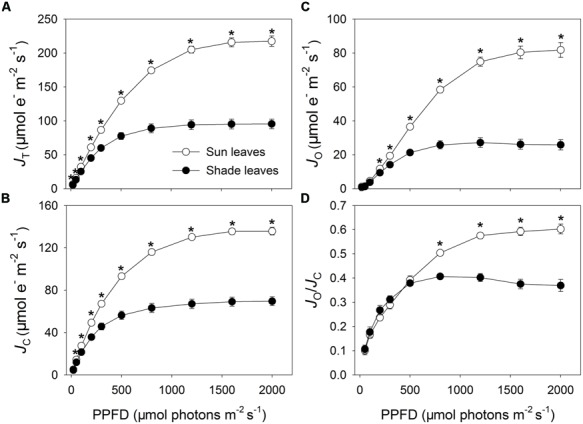
**Responses of total photosynthetic electron flow through PSII (*J*_**T**_, **A**), electron flow devoted to RuBP carboxylation (*J*_**C**_, **B**), electron flow devoted to RuBP oxygenation (*J*_**O**_, **C**), and *J*_**O**_/*J*_**C**_ (**D**) to incident PPFD for sun and shade leaves of tobacco.** Values are means ± SE (*n* = 4). Significant differences between leaf types (indicated by asterisks) were examined by independent *t*-test (*P* < 0.05).

The *J*_T_ through PSII was significantly higher in the sun leaves when light intensities were above 200 μmol photons m^-2^ s^-1^ (*P* < 0.0001; **Figure 2A**). Maximum values for *J*_T_ were 217 and 95 μmol electrons m^-2^ s^-1^ in sun and the shade leaves, respectively. Similarly, significantly more electron flow was devoted to RuBP carboxylation and oxygenation in the sun leaves, with maximum values of 135 and 69 μmol electrons m^-2^ s^-1^ for *J*_C_ (*P* < 0.0001; **Figure 2B**), and 82 and 26 μmol electrons m^-2^ s^-1^ for *J*_O_ (*P* < 0.0001; **Figure 2C**) in sun and shade leaves, respectively. We found it interesting that, at light intensities above 800 μmol photons m^-2^ s^-1^, the sun leaves had significantly higher *J*_O_/*J*_C_ ratios than did the shade leaves (*P* < 0.0001; **Figure 2D**); maximum ratios were 0.6 (sun) and 0.4 (shade). These results demonstrated that capacity by the photorespiratory pathway was enhanced in the sun leaves.

**FIGURE 3:**
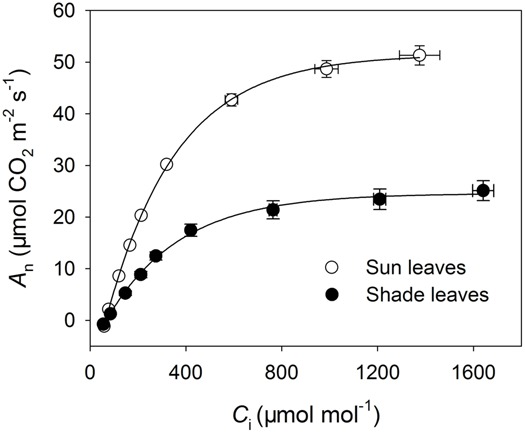
**Response of *A*_**n**_ to incident *C*_**i**_ in sun and shade leaves of tobacco.** Values are means ± SE (*n* = 4).

**FIGURE 4:**
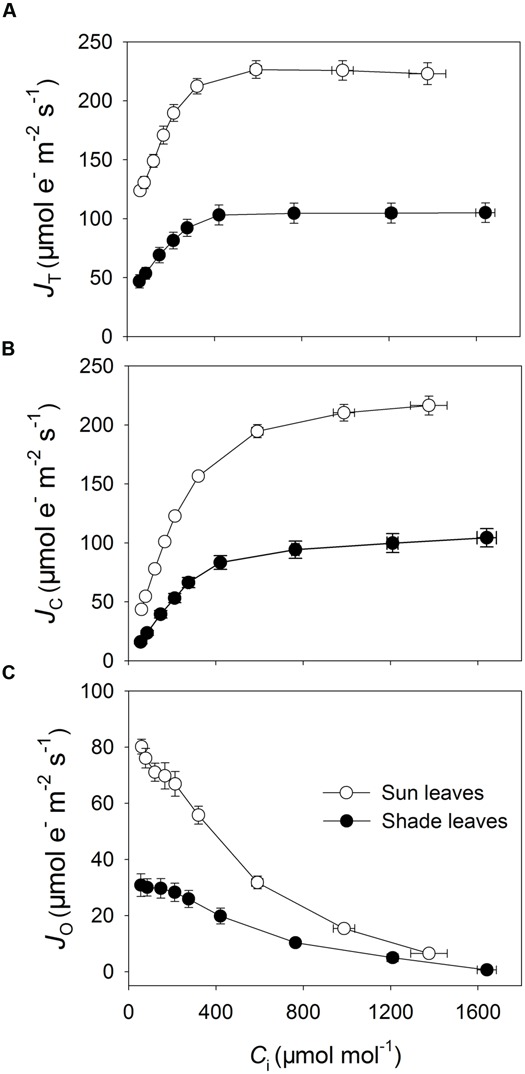
**Responses of total *J*_**T**_ through PSII (**A**), electron flow devoted to RuBP carboxylation (*J*_**C**_, **B**), and electron flow devoted to RuBP oxygenation (*J*_**O**_, **C**) to incident *C*_**i**_ in sun and shade leaves of tobacco.** Values are means ± SE (*n* = 4).

The *A*/*C*_i_ curves indicated that sun leaves had higher rates of CO_2_ assimilation when *C*_i_ was higher than 90 μmol mol^-1^ (**Figure 3**). The maximum photosynthetic rates in sun and shade leaves were 51.3 and 25.1 μmol CO_2_ m^-2^ s^-1^, respectively (**Figure 3**). Values for *J*_T_ and *J*_C_ were largely higher in the sun leaves under any CO_2_ concentration (**Figures 4A,B**), and they rose rapidly in parallel with *C*_i_ when it was below 300 μmol mol^-1^ (**Figures 4A,B**). When *C*_i_ was higher than 400 μmol mol^-1^, *J*_T_ was hardly increased in either type of leaf while *J*_C_ increased only slightly. As *C*_i_ rose, *J*_O_ gradually declined in both sun and shade leaves. However, *J*_O_ values were always higher in the sun leaves, especially when the CO_2_ concentration was low. For example, at a *C*_i_ of 55 μmol mol^-1^, *J*_O_ was 80 and 31 μmol electrons m^-2^ s^-1^ in sun and shade leaves, respectively (**Figure 4C**). At low *C*_i_, the affinity of Rubisco to O_2_ was markedly increased, and operation of the photorespiratory pathway consumed most of the products of linear electron flow. Consequently, *J*_O_ was maintained at a high level under low-*C*_i_ conditions (**Figure 4C**). When *C*_i_ was above 1200 μmol mol^-1^, the affinity of Rubisco to CO_2_ was largely increased, such that operation of the Calvin cycle consumed most of the products of linear electron flow. Thus, *J*_O_ was maintained at a low level when the *C*_i_ was elevated (**Figure 4C**).

**FIGURE 5:**
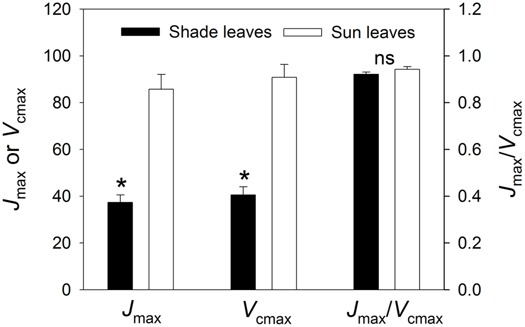
**Maximum rates of RuBP regeneration (*J*_**max**_) and RuBP carboxylation (*V*_**cmax**_), as well as *J*_**max**_/*V*_**cmax**_ ratios for sun and shade leaves.** Values are means ± SE (*n* = 4). Both *J*_max_ and *V*_cmax_ were significantly higher in sun leaves. The *J*_max_/*V*_cmax_ ratio did not differ significantly between sun and shade leaves. Significant differences between leaf types (indicated by asterisks) were examined by independent *t*-test (*P* < 0.05).

At 24°C, *J*_max_ was 86 for sun leaves and 37 μmol m^-2^ s^-1^ for shade leaves, while respective *V*_cmax_ values were 91 and 41 μmol m^-2^ s^-1^ (**Figure 5**). Sun leaves had significantly higher values for both *J*_max_ and *V*_cmax_ (*P* < 0.0001). The latter component is linearly and positively correlated with Rubisco content (Yamori et al., 2010a). Accordingly, we also established that sun leaves had a higher Rubisco content. Corresponding *J*_max_/*V*_cmax_ ratios were 0.94 for sun leaves and 0.92 for shade leaves. The lack of any significant difference in *J*_max_/*V*_cmax_ ratio between leaf types indicated that the ratio of electron transport capacity to Rubisco activity did not vary between them.

**FIGURE 6:**
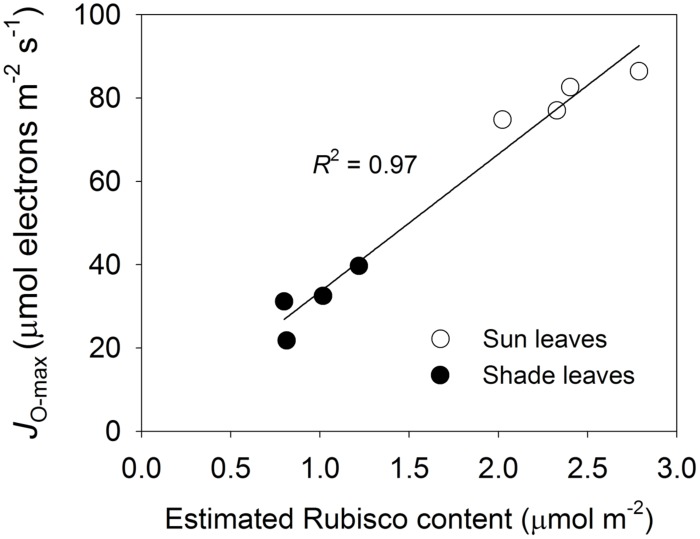
**Relationship between estimated Rubisco content and *J*_**O-max**_ for sun and shade leaves, as estimated from *A*-*C*_**i**_ curves developed at 24°C and a saturating light of 1200 μmol photons m^-**2**^ s^-**1**^.** Regression equation shown in figure is *y* = 33.03*x* + 0.47.

**FIGURE 7:**
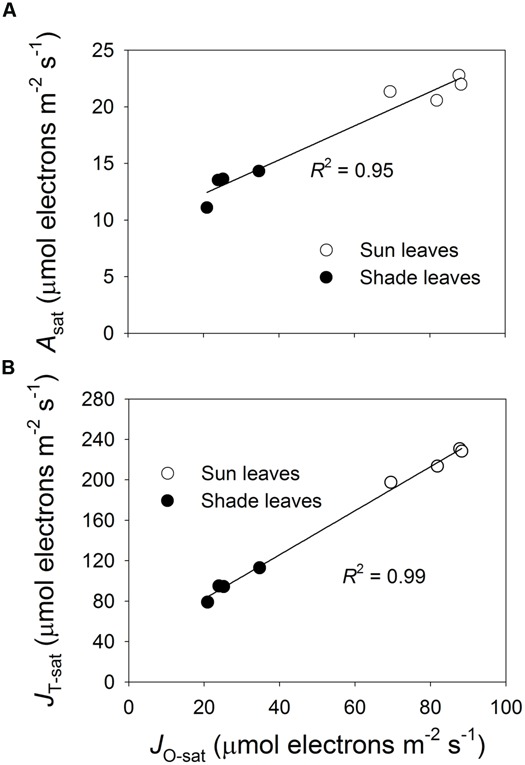
**Relationship between *J*_**O-sat**_ and *A*_**n-sat**_**(A)** and *J*_**T-sat**_**(B)** for sun and shade leaves.** Values were estimated from light response curves developed at 24°C. Values for *J*_O-sat_, *A*_n-sat_, and *J*_T-sat_ represent *J*_O_, *A*_n_, and *J*_T_, respectively, under saturating light intensity of 2000 μmol photons m^-2^ s^-1^. Regression equations are *y* = 0.15*x* + 9.3 **(A)** and *y* = 2.18*x* + 38.82 **(B)**.

The relationship between estimated Rubisco content and *J*_O-max_ was strong and linear, based on the *A*/*C*_i_ curves developed at 1200 μmol photons m^-2^ s^-1^ and 24°C (**Figure 6**). This indicated that the capacity of the photorespiratory pathway was coordinated with the level of Rubisco. Our light response curves at 24°C also demonstrated strong linear relationships among light-saturated *J*_O_ (*J*_O-sat_), light-saturated photosynthesis (*A*_sat_), and light-saturated *J*_T_ (*J*_T-sat_; **Figure 7**). These results suggested that high photosynthetic capacity in the sun leaves was accompanied by enhanced capacity for the photorespiratory pathway.

## DISCUSSION

### GROWTH LIGHT INTENSITY INFLUENCES THE CAPACITY OF THE PHOTORESPIRATORY PATHWAY

Light response curves revealed that the sun leaves had greater electron flow for photorespiration under high light when *C*_a_ was 400 μmol mol^-1^ (**Figure 2C**). Moreover, when exposed to high light intensities above 800 μmol photons m^-2^ s^-1^ and at a *C*_a_ of 400 μmol mol^-1^, the *J*_O_/*J*_C_ ratio was significantly higher for sun leaves (**Figure 2D**). The *A*/*C*_i_ curves indicated that, under strong illumination, sun leaves also had higher electron flow for photorespiration associated with low *C*_i_. These results demonstrated that sun leaves have improved capacity for the photorespiratory pathway when compared with shade leaves.

Photorespiration begins with the oxygenation of RuBP catalyzed by Rubisco. The affinity of Rubisco is mainly affected by temperature and *C*_i_ (von Caemmerer, 2000). Because our sun and shade plants were grown in the same phytotron at 24°C, the difference in Rubisco affinity was mainly related to *C*_i_. The higher *A*_n_ in sun leaves also led to lower *C*_i_ under more intense light. This reduction in *C*_i_ increased the affinity of Rubisco for O_2_. Furthermore, as indicated by values for *V*_cmax_, the sun leaves had a higher Rubisco content than did the shade leaves (Yamori et al., 2010a). Consequently, the rate of RuBP oxygenation was much higher in the sun leaves. The oxygenation of RuBP produces glycolate-2-phosphate, which then inhibits enzymes in the Calvin Cycle that are involved in regenerating RuBP (Anderson, 1971). Even low levels of glycolate-2-phosphate synthesis are detrimental to plants when this compound or other intermediates of the photorespiration process are accumulated (Peterhansel and Maurino, 2011). To avoid the harmful effects of glycolate-2-phosphate and other photorespiratory intermediates such as glycine and glyoxylate (Chastain and Ogren, 1989; Campbell and Ogren, 1990; Hausler et al., 1996; Eisenhut et al., 2007), the photorespiratory pathway must be accelerated in the sun leaves. Up-regulation of glycine decarboxylase can improve the rate of photosynthesis by decreasing the glycine content (Timm et al., 2012). Therefore, the increased capacity of the photorespiratory pathway that we found in our sun leaves probably prevented the accumulation of glycolate-2-phosphate and other photorespiratory intermediates, contributing in part to the maintenance of a high rate of photosynthesis.

The photorespiratory pathway is a complex process that depends on many enzymes involved in carbon and nitrogen metabolism, such as ferredoxin-dependent Glu, Ser hydroxymethyltransferase, Glu/malate transporter, glycerate kinase, and glycine decarboxylase (Somerville and Ogren, 1980, 1981, 1983; Somerville and Somerville, 1985; Blackwell et al., 1990; Wingler et al., 1997, 1999; Boldt et al., 2005). Because this pathway involves several cellular components, transporters responsible for supplying those enzymes are essential for photorespiratory processes (Eisenhut et al., 2013; Weber and Bauwe, 2013). Recently, the plastidial glycolate/glycerate transporter, PLGG1, was identified (Pick et al., 2013). Compared with shade leaves, sun leaves probably have increased synthesis of those enzymes and transporters, which then accelerates operation of the photorespiratory pathway.

### THE ROLE OF THE PHOTORESPIRATORY PATHWAY IN REGULATING CO_2_ ASSIMILATION

The estimated Rubisco content was positively correlated with the capacity of the photorespiratory pathway in our experiments. Under *C*_a_, the higher Rubisco content and lower *C*_i_ in the sun leaves induced a higher rate of RuBP oxygenation compared with the shade leaves. The greater capacity of photorespiratory pathway enhanced RuBP regeneration. Therefore, the positive relationship between the estimated Rubisco content and *J*_O-max_ suggested that RuBP oxygenation and regeneration are balanced via the photorespiratory pathway.

The limiting step for CO_2_ assimilation in leaves is mainly affected by the *J*_max_*/V*_cmax_ ratio (Yamori et al., 2010a,b, 2011). Although our shade leaves had significantly lower values for *J*_max_ and *V*_cmax_, their ratio did not differ from that of the sun leaves. According to the photosynthetic model of Yamori et al. (2011), the low *J*_max_*/V*_cmax_ ratio means that the *A*_n_ in sun and shade leaves is mainly limited by RuBP regeneration. Two pathways exist in C_3_ plants for RuBP regeneration: (1) recycling of glycerate-3-phosphate into RuBP wholly through the Calvin Cycle, and (2) recycling of glycolate-2-phosphate into glycerate-3-phosphate and then into RuBP through the photorespiratory pathway and the Calvin Cycle. The first pathway is rapid and is completed in the chloroplasts. However, the second is relative slow and involves three organelles – the chloroplasts, mitochondria, and peroxisomes (Takahashi et al., 2007; Timm et al., 2012). Thus, the capacity of the photorespiratory pathway tends to be a rate-limiting step for RuBP regeneration in both sun and shade leaves when exposed to *C*_a_ and strong illumination.

This pathway capacity can control C_3_ photosynthesis (Timm et al., 2012), because, if it is as low in the sun leaves as in the shade leaves, the rate of RuBP oxygenation will greatly exceed the rate of RuBP regeneration in the sun leaves. Thus, the Calvin Cycle in the sun leaves might subsequently become restricted by a lack of RuBP. To maintain a steady and high *A*_n_, RuBP oxygenation and regeneration must be balanced. For sun leaves, enhancement of the photorespiratory pathway can accelerate the rate of RuBP regeneration and helps maintain a high *A*_n_. These results, therefore, allow us to conclude that increasing the capacity of the photorespiratory pathway is an important strategy by which sun leaves from tobacco can maintain a high *A*_n_ at *C*_a_.

Although CO_2_ is released in the mitochondria through functioning of the photorespiratory pathway, C_3_ plants trap the photorespirated and respired CO_2_ within single mesophyll cells (Busch et al., 2013). This trapping should then lead to a rise in chloroplast CO_2_ concentrations and, ultimately, improve the specificity of Rubisco to CO_2_. Consequently, C_3_ plants improve their rates of photosynthesis by re-assimilating photorespirated CO_2_ (Busch et al., 2013). In sun leaves, a higher rate of RuBP oxygenation and acceleration of the photorespiratory pathway can increase the rate at which this photorespiratory CO_2_ is released, thereby raising the *A*_n_.

### THE ROLE OF THE PHOTORESPIRATORY PATHWAY IN REGULATING PHOTOSYNTHETIC ELECTRON FLOW

In higher plants, photosynthetic electron transfer from PSII to PSI converts light energy into ATP and NADPH, which is regulated by the proton gradient between the thylakoid membrane and stroma (ΔpH; Tikkanen and Aro, 2014). The generation of ΔpH is dependent on (1) the accumulation of protons in the lumen from both the water-splitting reaction of PSII and electron transfer via Cyt*b*_6_/*f*, and (2) the rate of proton eﬄux from the lumen to stroma via ATP synthase. The energy transfer efficiency from light harvesting complex to the photosystems is enhanced by a decrease in ΔpH but reduced by an increase in ΔpH (Tikkanen and Aro, 2014). The Cyt *b*_6_/*f* complex couples electron transfer to proton transfer, which is controlled by ΔpH. The higher the ΔpH, the slower that electrons can be transferred from PSII to PSI via Cyt *b*_6_/*f* (Tikkanen and Aro, 2014). When the photorespiratory pathway is up-regulated by over-expression of the H-protein of glycine decarboxylase, photosynthetic electron transport from PSII to PSI must increase (Timm et al., 2012). Otherwise, once photorespiratory pathway becomes impaired, the ΔpH rises and causes electron transfer from PSII to PSI to be suppressed (Takahashi et al., 2007). Here, enhancement of photorespiratory pathway in the sun leaves accelerated the consumption of ATP and NADPH, thus decreasing ΔpH and favoring the *J*_T_ from PSII to PSI (**Figure 7B**). Taken together, we concluded that the higher capacity of the photorespiratory pathway in sun leaves regulated the ΔpH and then accelerated electron transfer.

## CONCLUSION

Our results strongly indicate that tobacco leaves grown under stronger irradiance have a higher rate of RuBP oxygenation compared with leaves exposed to low light levels. This is due to higher Rubisco contents and diminished *C*_i_. Meanwhile, the capacity of the photorespiratory pathway is improved in plants grown under a high light intensity, which enables them to hasten the recycling of glycolate-2-phosphate into glycerate-3-phosphate. This then regulates the balance between RuBP oxygenation and regeneration and helps to modulate the RuBP content in chloroplasts. Therefore, enhancement of photorespiratory pathway is essential for sun leaves to maintain a high *A*_n_.

## AUTHOR CONTRIBUTIONS

Wei Huang, Shi-Bao Zhang, and Hong Hu: Conceived and designed the experiments. Wei Huang: Performed the experiments. Wei Huang and Shi-Bao Zhang: Analyzed the data. Wei Huang and Shi-Bao Zhang: Contributed reagents/materials/analysis tools. Wei Huang, Shi-Bao Zhang, and Hong Hu: Contributed to the writing of the manuscript.

## Conflict of Interest Statement

The authors declare that the research was conducted in the absence of any commercial or financial relationships that could be construed as a potential conflict of interest.

## ABBREVIATIONS

*A*_n_: rate of CO_2_ assimilation
*C*_a_: atmospheric CO_2_ concentration
*C*_i_: intercellular CO_2_ concentration
*g*_s_: stomatal conductance
*J*_C_: electron flow devoted to carboxylation of RuBP
*J*_max_: maximum rate of RuBP regeneration
*J*_O_: electron flow devoted to oxygenation of RuBP
*J*_T_: photosynthetic electron flow
Rubisco: ribulose-1,5-bisphosphate carboxylase/oxygenase
RuBP: ribulose- 1,5-bisphosphate
*V*_cmax_: maximum rate of RuBP carboxylation.
